# Cochlear dysfunction and microvascular complications in patients with type 1 diabetes mellitus

**DOI:** 10.1186/s13098-018-0380-z

**Published:** 2018-11-09

**Authors:** João Soares Felício, Lilian de Souza d’Albuquerque Silva, Carlliane Lima e Lins Pinto Martins, João Felício Abrahão Neto, Manuela Nascimento de Lemos, Fabrício de Souza Resende, Wanderson Maia da Silva, Angélica Leite de Alcântara, Maria Clara Neres Iunes de Oliveira, Norberto Jorge Kzan de Souza Neto, Isabela Imbelloni Farias de Franco, Nathalie Abdallah Zahalan, Luísa Correa Janaú, Ana Carolina Contente Braga de Souza, Flavia Marques Santos, Natércia Neves Marques de Queiroz, Neyla Arroyo Lara Mourão, Márcia Costa dos Santos, Karem Miléo Felício, Franciane Trindade Cunha de Melo

**Affiliations:** Endocrinology Division - Programa de Pós-Graduação em Oncologia e Ciências Médicas, University Hospital João de Barros Barreto, Federal University of Pará, Mundurucus Street, 4487, Guamá, Belém, PA Postal Code: 66073-000 Brazil

**Keywords:** Type 1 diabetes mellitus, Cochlear dysfunction, Sensorineural hearing loss, Otoacoustic emissions, Diabetic kidney disease, Cardiac autonomic neuropathy

## Abstract

Sensorineural hearing impairment has been associated with DM, and it is probably linked to the same pathophysiological mechanisms as well-established in microvascular diabetes complications. The study of otoacoustic emissions (OAEs) is useful to identify subclinical cochlear dysfunction. Therefore, the aim of this study was to evaluate the association between abnormal OAEs responses, diabetic kidney disease (DKD) and diabetic cardiac autonomic neuropathy (CAN). We performed a cross-sectional study with 37 type 1 DM patients without auditory symptoms, submitted to the study of Distortion Product Otoacoustic Emissions (DPOAEs) and screened for DKD and CAN. The otoacoustic emissions responses were considered abnormal in 27/37 (73%) patients. A correlation was found between abnormal OAEs responses and presence of DKD (r = 0.36, p < 0.05), and 14/16 (88%) patients with a lower amplitude of OAEs in 8 kHz frequency band presented DKD. Abnormal OAEs responses in the 6 kHz frequency band were correlated with the presence (r = 0.41, p = 0.01) and severity of CAN (r = 0.44, p < 0.001). Additionally, 7/9 (78%) patients with abnormal OAE responses in this frequency also presented abnormal CAN scores. Our results suggest that abnormal otoacoustic emissions responses in high frequency bands are associated with diabetes microvascular complications and could be a risk marker for DKD and CAN, presenting low sensitivity and high specificity. Therefore, assuming that hearing impairment is a pre-clinical stage of hearing loss, performing distortion product otoacoustic emissions in T1DM patients with microvascular complications could be useful to identify those who would be benefit with regular audiologic follow up and tighter diabetes control.

## Introduction

An association between diabetes mellitus (DM) and sensorineural hearing impairment has been widely discussed, but despite a large number of studies performed, it remains controversial [1–5]. Hearing loss is very prevalent among the elderly population, however it is almost twice as common in diabetic adults [6]. Several studies also demonstrated a high prevalence of hearing impairment in diabetic patients, suggesting that DM may be an independent risk factor for hearing loss, justifying screening for this condition in these patients [6–9].

It is well known that DM is associated to many microvascular complications. Human cochlea has an extensive microvasculature and it is considered vulnerable to the effects of microangiopathy, one of the hyperglycemia consequences. Hearing loss in DM may be a result of microangiopathy and several studies postulate this [10–12]. The study of otoacoustic emissions (OAEs) is a simple, objective, highly reproducible, frequency-specific and noninvasive method for the evaluation of the cochlear micromechanics [13, 14], and is very useful to identify subclinical sensorineural hearing impairment [15, 16], which is more frequently found by distortion product of otoacoustic emissions (DPOAE) in diabetic patients [17]. The aim of the present study is to evaluate the association between abnormal otoacoustic emissions responses, DKD and CAN in patients with type 1 diabetes mellitus (T1DM) without auditory symptoms.

## Methods

### Study design and patients

We performed a cross-sectional study in 37 patients with T1DM. Age under 8 years old, patients with any acute or chronic otological pathology, personal or family history of hearing loss, use of ototoxic drugs, excessive exposure to noise, presence of infection or other urinary tract diseases and any known cardiac diseases were exclusion criterias. This study was developed according to Declaration of Helsinki and the Nuremberg Code and was approved by the University Hospital João de Barros Barreto ethics committee, protocol number 236/08. Signed consent was obtained from all patients.

### Clinical and laboratory data

Medical records analysis and physical examination were performed. Information on demographics, physical measures, pre-existing clinical conditions, duration of T1DM (in years), current medications and body mass were analyzed. Laboratory tests were also performed and included measurements of fasting plasma glucose (FPG), total cholesterol, LDL cholesterol, HDL cholesterol, triglycerides, thyroid-stimulating hormone (TSH), free thyroxine (FT4), serum creatinine and glycated hemoglobin (HbA1c) by high-performance liquid chromatography method (HPLC). GFR was calculated using and modification of diet in renal disease (MDRD) equations.

In addition, we evaluated the presence of DKD, obtaining at least three 24-h urine samples for measuring albuminuria (by immunoturbidimetry). After these initial procedures, all patients underwent routine otorhinolaryngological evaluation with otoscopy, followed by acoustic reflexes assessment, vocal and pure tone audiometry, tympanometry and Distortion Product Otoacoustic Emissions (DPOAEs). We have excluded from our analysis any patient who had current or medical history of otological problems. Additionally, the subjects were screened for CAN.

### Otoacoustic emissions

OAEs are sounds caused by the motion of the cochlea’s sensory hair cells as they energetically respond to auditory stimulation, they are recorded by a probe containing a microphone transducer inserted into the ear canal. Separate responses from different parts of the cochlea are obtained by splitting the response into frequency bands. Therefore, otoacoustic emissions are frequency-specific responses and tend to emerge only in frequency bands in which hearing is near normal, providing a useful pointer to normally and abnormally functioning parts of cochlea [18].

The method of OAEs recording used in this study was the Distortion Product Otoacoustic Emissions (DPOAEs), which uses tonal stimulation, with a wider frequency range observation and wide-spread clinical use [18, 19].

The frequencies analyzed in this study were 0.5 k, 1 k, 2 k, 4 k, 6 k and 8 k Hz. The test was considered abnormal when the signal-to-noise ratio (SNR) was below 6 dB in at least one of the frequencies assessed [20].

### Diabetic kidney disease

DKD was diagnosed after we evaluated the presence of micro or macroalbuminuria in at least two of the three 24-h urine samples, according to ADA recommendation [21]. Microalbuminuria was defined as a urinary albumin excretion rate (UAER) in the range of 30–300 mg/24 h, and macroalbuminuria, as UAER equal to or greater than 300 mg/24 h. However, nondiabetic renal disease has been significantly found in diabetic patients, either alone or combined with DKD, in a recent study [22]. Therefore, our data should be analyzed carefully. Additionally, we performed urinalysis, urine culture and an ultrasound scan of the urinary tract to evaluate the presence of infection or other urinary tract conditions that might interfere with the study results.

### Diabetic cardiac autonomic neuropathy

Tests were conducted in both groups as proposed by Ewing et al. [23] and Valensi et al. [24]. They were based on the response of the heart to the Valsalva manoeuvre (Valsalva ratio), variation in heart rate (RR interval) during deep breathing (deep breathing test) and response of BP to the change recumbent from lying position to standing position (lying-to-standing test). The first two tests reflect parasympathetic integrity, and the last reflects sympathetic integrity.

#### Deep breathing test

Increases in heart rate (RR interval) while breathing were measured during the deep breathing test. The heart rate usually depends on parasympathetic nervous system integrity. Patients with diabetic CAN have a considerable reduction or even complete absence of increases in heart rate. Patients stayed in the supine position, were quiet and took six deep breaths in 1 min (5 s for inspiration and 5 s for expiration) while an electrocardiogram was recorded, using a marker to indicate the end of each inspiration and expiration. Increases in heart rate during the breathing were recorded (normal heart rate increase was defined as at least 15 beats/min, ‘borderline’ as 11 beats/min to 14 beats/min, and abnormal as 10 beats/min or less) [25].

Valsalva ratio (cardiac response to valsalva manoeuvre): During the effort period, BP falls and the heart rate should rise. After resting, the BP rises, overtaking its normal rest value, and the heart rate decreases. The test consisted of forced exhalation and maintaining a pressure of 40 mmHg for 15 s while an electrocardiogram was recorded. It was performed three times in the space of 1 min, with the patient resting between each test. The results were expressed as a Valsalva ratio, which is the ratio between the highest RR interval after the manoeuvre (reflecting bradycardia following the relaxation) and the lowest RR interval during the manoeuvre (which reflects tachycardia during exercise). The test was performed three times and the mean value for the ratio was used. Valsalva ratios of 1.21 or greater were defined as normal, 1.11–1.20 as ‘borderline’ and 1.10 or less as abnormal.

#### Lying-to-standing test

This test was performed analysing variation in blood pressure from lying to standing positions, as previously described. Decreases > 20 mmHg in systolic blood pressure were considered abnormal [23, 24, 26]. Reproducibility of these methods has been demonstrated in diabetic patients. The coefficients of variation are 9.2%, 12.6% and 6.4% for the Valsalva manoeuvre, deep breathing test and lying-to standing test, respectively [27].

For statistical analysis, the severity of CAN was measured by a score assigned to each patient according to the results obtained in the tests, as follows: 0—all tests resulted in normal values; 1—only one test resulted in abnormal values; 2—two tests resulted in abnormal values; and 3—all tests resulted in abnormal values. Individuals with test results classified as borderline received a score of 0.5 for the corresponding test. One abnormal test or two borderline results in different tests were considered as CAN for statistical analysis.

### Statistical analysis

Categorical variables were described as frequency (percentage). Numeric variables with normal distribution were described as mean (SD) and non-normally distributed as median (minimum–maximum). Chi square and Fisher’s exact test were used to compare categorical variables. The Student’s t-test and Mann–Whitney test were used to compare subgroups with and without normal distributions, respectively. To establish correlations between variables, Pearson and Spearman tests were used. The analysis of variance compared more than two subgroups with normal distribution, and the Kruskal–Wallis test was used to compare more than two subgroups without normal distribution. Sensitivity, specificity and accuracy to the cut-off points previously established were estimated based on screening test. The best cut-off point was defined based on the Youden Index (J) and, additionally, a ROC (Receiver Operating Characteristic) Curve was constructed. The cut-off with maximum sensitivity and specificity in the ROC curve was defined as the minimum value in the equation [(1 − sensitivity)^2^ + (1 − specificity)^2^] and the accuracy was estimated based on the area under the ROC curve. Predictive values and likelihood ratios were also calculated from the values of sensitivity and specificity. The ROC curve was built with 8 kHz frequency bands in dB and the presence or not of DKD, based on ADA criteria. All tests were performed using the SPSS^®^ statistics software. Further, the results were considered significant if p-value ≤ 0.05.

Interferences are represented by hypothesis tests with a bilaterally significance level of 0.05. All information was stored and processed with the software SigmaStat (Jandel Scientific) version 3.5 and SPSS (Statistical Package for Social Sciences) 22.0 (IBM).

### Patient and public involvement

Our research question was developed based on previous studies that have already found auditory damage as a DM complication, related to microvascular physiopathology. Furthermore, patients were included in this study as samples, and were not involved in recruitment and conduct of this study. Informed consent was obtained from all of them and they had access to the results of this research. T1DM patients who had abnormal OAE responses are still being followed in our center with special attention in diabetic kidney disease and cardiac autonomic neuropathy.

## Results

The clinical and laboratory data of the patients are described in Table 1. Three patients presented hypertension (7.7%), 5 had dyslipidemia (12.8%) and 3 presented hypothyroidism (7.7%). The individuals with hypothyroidism were using a stable dose of levothyroxine and had normal thyroid function. All patients were in insulin therapy and 7 (17.9%) also used metformin. Only one patient reported smoking.

**Table 1:** Clinical and laboratorial data of patients with T1DM

Variables	
Age (years)	23 ± 8
Sex (F/M)	20/17 (54/46%)
Duration of diabetes (years)	10.2 (2 to 24)
HbA1c (%)	9.4 ± 2.5
SBP (mmHg)	109 ± 14
DBP (mmHg)	71 ± 10
BMI (kg/m^2^)	23 ± 3
Total cholesterol (mg/dl)	185 ± 63
HDL cholesterol (mg/dl)	49 ± 13
LDL cholesterol (mg/dl)	112 ± 51
Triglycerides (mg/dl)	115 ± 78

HbA1c, glycated hemoglobin; SBP, systolic blood pressure; DBP, diastolic blood pressure; BMI, body mass index

### Otoacoustic emissions and audiometry

Only 10/37 (27%) patients had normal Distortion Product Otoacoustic Emissions in all frequency bands. The OAEs responses were considered abnormal in 27/37 (73%) patients.

**Fig. 1:**
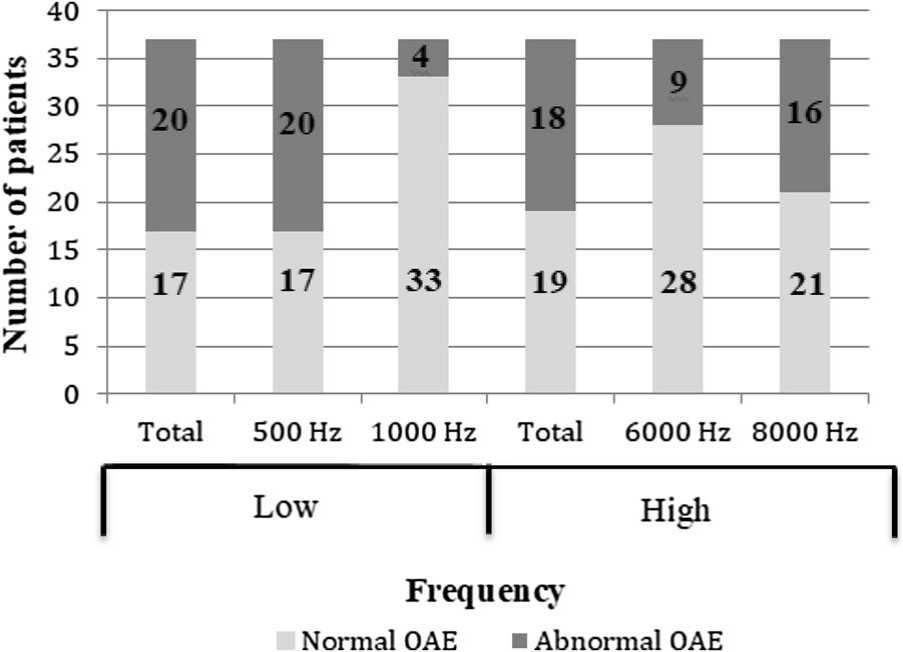
Frequency-specific distribution of abnormal OAEs in T1DM patients

The frequency-specific responses are presented in Fig. 1. Fourteen patients had abnormalities in two or more frequency bands.

Abnormal audiometry was observed in 6/37 (16%) patients and it was correlated with the CAN score (r = 0.42, p < 0.05). There was no correlation between auditory impairment by audiometry and DKD.

### Otoacoustic emissions and DKD

DKD was observed in 21/37 (57%) patients and just two of them presented macroalbuminuria. GFR and UAER are described in Table 2.

**Table 2:** Renal function and albuminuria in T1DM patients (n = 37)

	Normoalbuminuria (N = 16)	Microalbuminuria (N = 17)	Macroalbuminuria (N = 4)	p
Serum creatinine (mg/dl)	0.9 ± 0.1	0.9 ± 0.2	1.0 ± 0.2	NS
GFR (ml/min/1.73 m^2^)	109.4 ± 20.3	94.4 ± 23.6	81.7 ± 16.6	< 0.05^†^
Albuminuria (mg/24 h)	23.8 ± 8.2	51.8 ± 25.7	688.6 ± 857.0	< 0.05^‡^

^†^ p < 0.05 between normoalbuminuric versus micro and macroalbumiunric patients

^‡^p < 0.05 between all groups

Abnormal OAEs responses were correlated with the presence of DKD (r = 0.36, p < 0.05) and UAER (r = 0.34, p < 0.05). We have found UAER of 96 ± 137 and 157 ± 404 mg/24 h in patients with normal and abnormal otoacoustic emissions responses, respectively; however, it was not statistically significant.

**Fig. 2:**
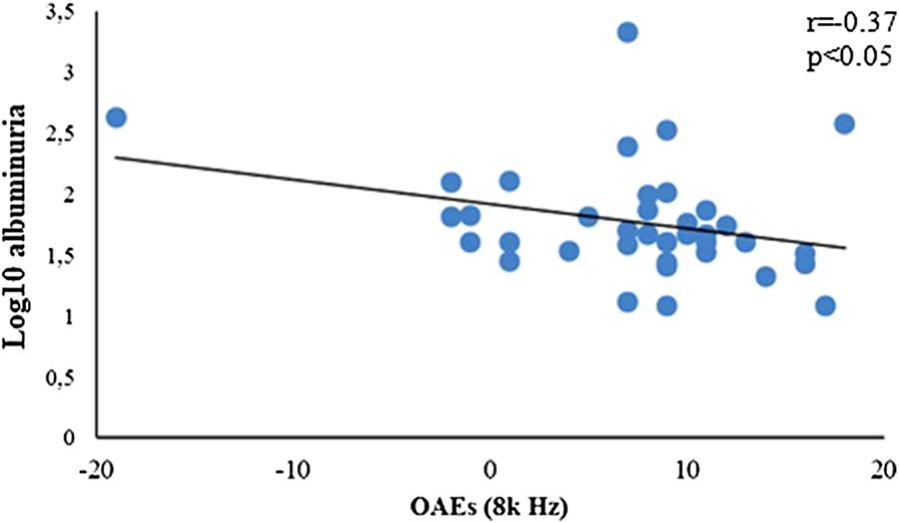
Correlation between albuminuria and otoacoustic emissions (OAEs) in the frequency band of 8000 Hertz in patients with T1DM (n = 37)

Additionally, we have observed a correlation between the SNR of OAEs in the 8 k Hz frequency band and UAER (r = − 0.37, p < 0.05) (Fig. 2). Moreover, among the 16 patients with abnormal otoacoustic emissions responses in this frequency band, 14 (88%) presented DKD.

As screening test to DKD, in 8 k Hz frequency band, the study of Distortion Product Otoacoustic Emissions showed 30% of sensitivity and 85% of specificity, with positive and negative predictive values of 90% and 22%, respectively. Test accuracy was 40%.

**Fig. 3:**
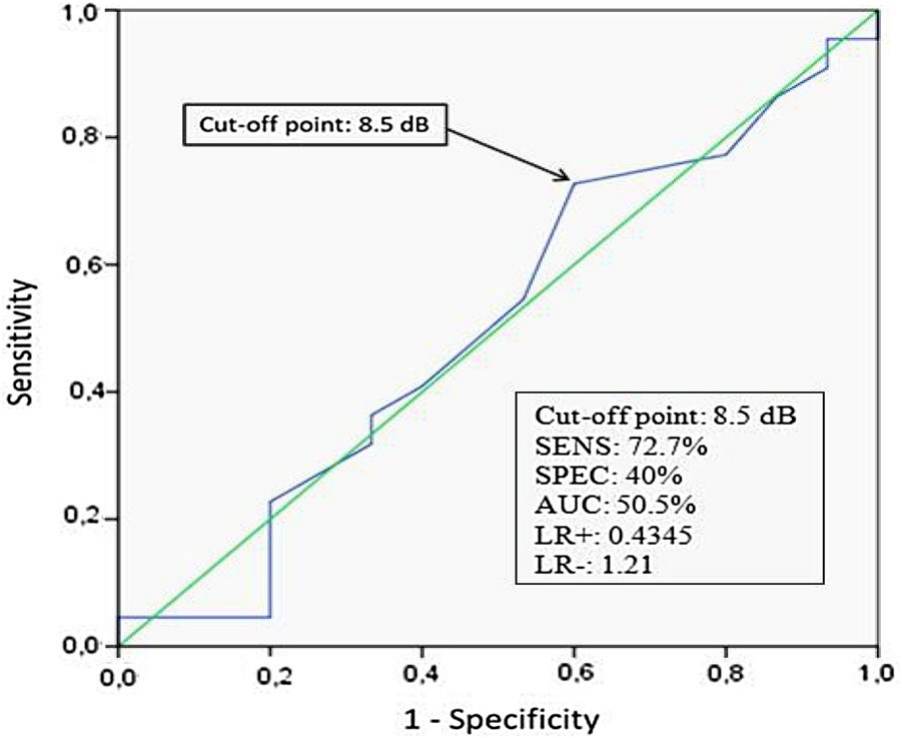
ROC curve and cut-off point for the Signal Noise Ratio (SNR) and presence of DKD. Sens, sensitivity; Spec, specificity; AUC, area under the curve; LR+, positive likelihood ratio; LR−, negative likelihood ratio

Normal values to otoacoustic emissions are already well established (SNR < 6 dB), so our aim was not to identify a better cut-off point, but to find the relation between DKD and otoacoustic emissions. In this case, we have done a ROC curve and added this analysis in Fig. 3. The best cut-off in decibels was 8.5 dB (above literature standard) in 8.000 Hz frequency band, which showed 73% of sensitivity and 40% of specificity, with 50% of accuracy.

### Otoacoustic emissions and autonomic neuropathy

The results of the autonomic function tests are shown in Table 3. CAN was diagnosed in 17/37 (46%) patients. Valsalva test was abnormal in 2/37 (6%), deep breathing test was in 10/37 (27%) and lying-to-standing test was normal (73%) or borderline (23%) in all patients. Five patients (13%) were classified as borderline in two different tests and were included in our analyses and diagnosed with CAN.

**Table 3:** Results of the autonomic function tests in T1DM patients (N = 37)

Autonomic function test	Normal	Borderline	Abnormal
Valsalva test	29/37 (78%)	6/37 (16%)	2/37 (6%)
Deep breathing test	24/37 (65%)	3/37 (8%)	10/37 (27%)
Lying-to-standing test	27/37 (73%)	10/37 (27%)	0/37

Abnormal OAEs responses in 6 k Hz frequency band were correlated with presence (r = 0.41, p = 0.01) and severity of CAN (CAN score) (r = 0.44, p < 0.001). Additionally, 7/9 (78%) of patients with abnormal OAEs responses in this frequency band also presented CAN.

Furthermore, otoacoustic emissions responses in 6 k Hz frequency band were correlated with results of Valsalva test (r = 0.47, p < 0.001), deep breathing test (r = 0.33, p < 0.05) and lying-to-standing test (r = 0.32, p = 0.05).

Abnormal OAEs responses in low frequency bands (0.5 k and 1 k Hz) correlated with postural BP change in lying-to-standing test (0.5 k Hz: r = − 0.35, p < 0.05; 1 k Hz: r = − 0.42, p = 0.01).

As screening test to CAN, distortion product otoacoustic emissions showed 41% of sensitivity and 90% of specificity, with positive predictive value (PPV) of 78% and negative predictive value (NPV) of 64%. Test accuracy was 67%.

## Discussion

The present study demonstrated a high prevalence of abnormal OAE responses in T1DM patients, and also an association between this finding and presence and severity of DKD and CAN. This correlation was more evident in high frequency bands, which present greater reproducibility. In these frequencies, OAEs responses showed high specificity but low sensitivity to detection of DKD and CAN.

Several studies have demonstrated the presence of lower amplitude of OAEs in T1DM patients with normal auditory thresholds, pointing to early subclinical modifications of the inner ear caused by the metabolic changes of diabetes [28–30]. In OAEs assessment, higher frequencies (4 k, 6 k, and 8 k Hz) are considered the most specific for cochlear injury diagnosis [18] and are also the most frequently affected in the diabetes-related hearing impairment [3, 6, 10, 31, 32]. Ottaviani et al. [33], studying 60 T1DM patients without hearing symptoms, observed abnormal OAE responses in 28% of them, in spite of a normal audiometric hearing threshold. The higher prevalence of cochlear dysfunction observed in our sample may be at least in part justified by the worse glycemic control in our patients (HbA1c = 9.4 ± 2.6% vs 8.1 ± 1.8%). Another explanation involves the high prevalence of diabetes complications in our sample, given that we are a tertiary care hospital. As abnormal OAE responses are associated with microangiopathy, it is expected to find more cochlear dysfunction in a sample with high prevalence of diffuse microvascular complications. The association between abnormal otoacoustic emissions, DKD, and CAN, described in our study, reinforces that hypothesis. Therefore, if it is true that hearing impairment is a pre-clinical stage of hearing loss [34], performing distortion product otoacoustic emissions in T1DM patients with microvascular complications could be useful to prevent auditory damage. Given that, diabetic patients should be instructed about this damage and how to prevent it, aiming to improve glycemic control [35], avoid exposure to loud noises, ear infections and tympanic membrane perforations, which can jeopardize healthy hearing of these patients [36].

Sasso et al. [37], studying patients with type 2 diabetes mellitus, have not found association between optoacoustic emissions and DKD and diabetic retinopathy. It might have occurred because of the existence of many confounders in T2DM (older age, hypertension and the use of many concomitant ototoxic medications) that could interfere in audiometric evaluation. In addition, they have included patients with history of otological pathology. In our opinion, to avoid those confounders, the relation between diabetic complications and abnormal OAE should be better studied in type 1 diabetic patients.

The relationship between diabetes and hearing dysfunction has been well established [7, 32]. In a recent meta-analysis, Horikawa et al. found a high prevalence of hearing impairment in diabetic patients [7]. Moreover, a cross-sectional study including 5140 patients, showed an occurrence of hearing impairment in 54% of DM patients vs 32% in controls [32]. In addition, in other series, decreased distortion product otoacoustic emissions amplitudes in different frequency bands were associated with diabetic neuropathy [5, 38], nephropathy, and retinopathy [5]. However, these studies included people with type 2 DM, which is largely associated with other variables implicated in cochlear injuries, such as hypertension, dyslipidemia, and atherosclerosis [10, 39, 40].

Our data shows a DKD prevalence of 88% in patients with abnormal otoacoustic responses in 8 k Hz frequency band, that was additionally associated with albuminuria. In case of a disease with several differential diagnoses, it is important to use a specific method. Analyzing our ROC curve, the best cut-off point in decibels was 8.5 dB (above literature standard) in 8000 Hz frequency band, which showed 73% of sensitivity and 40% of specificity, with 50% of accuracy. Nevertheless, to question the current otoacoustic emissions normal threshold we would need a larger group. We have also found quite similar results between CAN and abnormal OAEs responses in 6 k Hz band. Some studies, using auditory brainstem evoked and audiometric techniques, have found a high risk of hearing impairment in T1DM patients [41, 42]. Lasagni et al. [43] observed that distortion product otoacoustic emissions intensities at medium frequencies (2.8–4 kHz) were significantly lower in T1DM, however, the study did not show an association between abnormal OAE responses and microvascular alterations, such as albuminuria and retinopathy. Nevertheless, Lisowska et al. were the only ones that assessed this issue in T1DM patients, using distortion product otoacoustic emissions in high frequencies. They performed a cohort study with 42 T1DM patients and have not found an association between DKD, retinopathy and abnormal otoacoustic emissions responses in high frequency bands [4]. This may have occurred because they have not searched OAEs at 8 k Hz frequency and no CAN screening was done. As we are aware, this is the first study to suggest an association between abnormal otoacoustic responses, especially in high frequency bands, and presence and severity of DKD and CAN in T1DM patients without auditory symptoms. However, our data should be interpreted carefully. Due to overlapping clinical features of glomerulopathies, DKD is often diagnosed in diabetic patients without further research. In 2013, Sharma et al. performed renal biopsy in 611 diabetic patients and found that only 227 (37%) had DKD alone, while 220 (36%) had nondiabetic renal disease solely, and 164 (27%) had both [22].

Retinopathy constitutes an independent risk marker for DKD, given its high sensitivity. Therefore, in diabetic patients with albuminuria but without retinopathy, renal biopsy is indicated to establish whether this laboratory finding can be attributed to diabetes [44]. In this scenario, performing distortion product otoacoustic emissions could help. Due to its high specificity, if it is abnormal in 8 k Hz band, the probability of DKD diagnosis is very high, so this procedure could be avoided. In fact, it is well established that loss of hair cells tend to occur in patients > 15 years of diabetes duration [45]; while DKD is typically developed after 10 years of T1DM diagnosis [27]. In other words, DM affects kidneys before cochlea, it could be the explanation why distortion product otoacoustic emissions is a highly specific but not too sensible test for DKD diagnosis. Larger studies are necessary to confirm this data.

Furthermore, in our study, 78% of the patients with a lower amplitude of OAEs in the 6 k Hz frequency band had abnormal autonomic function tests, suggesting that the otoacoustic emissions study in high frequencies also presents high specificity for the diagnosis of CAN. Friedman et al. [46] suggested that microangiopathy could be nonspecific rather than a causal finding, and Makashima and Tanaka [47] described atrophy of spiral ganglion neurons and demyelination of the auditory nerve in a few diabetic subjects. Demyelination is also the initial lesion in peripheral nerves of diabetic extremities, which could imply that hearing impairment can possibly be due to diabetic autonomic neuropathy of the auditory nerve. Di Leo et al. show in a study with 48 DM patients that OAE is reduced in patients with neuropathy compared to those without [48]. Therefore, one possible explanation is that autonomic neuropathy could be a via through which microvascular disease could lead to hearing impairment in diabetic patients.

Finally, the main limitation of our study was the small number of patients evaluated, although the analyzed frequencies are highly reproducible. Other weaknesses are the variables besides DM that could implicate in cochlear injuries, such as age, hypertension, dyslipidemia, smoking, excessive exposure to noise and use of ototoxic drugs. However, in our study, these confounders were minimum, since the presence of otological pathologies was an exclusion criterion, the population was young (average age of 23.5 years old), and there was a low prevalence of hypertension, dyslipidemia, and smoking.

## Conclusion

Our results suggest that abnormal otoacoustic emissions responses in high frequency bands are associated with diabetes microvascular complications and could be a risk marker for DKD and CAN, presenting low sensitivity and high specificity. However, this data should be confirmed in a larger sample. Therefore, assuming that hearing impairment is a pre-clinical stage of hearing loss [35], performing distortion product otoacoustic emissions in T1DM patients with microvascular complications could be useful to identify those who would be benefited with regular audiologic follow up and tighter glycemic control.

## Article summary

### Strengths and limitations of this study

#### Strengths

The great reproducibility of OAE responses in high frequency bands that showed high specificity to detection of DKD and CAN. In addition, confounders were minimum, since the presence of otological pathologies was an exclusion criterion, the population was young (average age of 23.5 years old), and there was a low prevalence of hypertension, dyslipidemia, and smoking

#### Limitations

The small number of patients evaluated and the possible presence of variables besides DM that could implicate in cochlear injuries, such as age, hypertension, dyslipidemia and smoking

## Abbreviations

T1DM: type 1 diabetes mellitus
OAE: otoacoustic emissions
DPOAE: distortion product otoacoustic emissions
DKD: diabetes kidney disease
CAN: cardiac autonomic neuropathy
ADA: American Diabetes Association
DM: diabetes mellitus
BMI: body max index
HPLC: high performance liquid chromatography
MDRD: modification of diet in renal disease
FPG: fasting plasma glucose
TSH: thyroid-stimulating hormone
FT4: free thyroxine
